# Unmet Needs and Behavioral Expressions: The Perspective of Individuals with Mild to Moderate Dementia

**DOI:** 10.1093/geroni/igaa057.3254

**Published:** 2020-12-16

**Authors:** Morgan Minyo, Katherine Judge

**Affiliations:** Cleveland State University, Cleveland, Ohio, United States

## Abstract

Research examining the illness experience from the perspective of individuals with dementia (IWDs) has gained increased attention. Previous studies suggest many IWDs experience unmet needs and behaviors that can impact their well-being. Most research has assessed these constructs using caregiver proxy-reports, resulting in underestimation of the amount and type of unmet needs. Additionally, no published studies have assessed self-reported behavioral expressions. As part of a larger study conducted in a memory care unit, the current pilot study examined the reliability of these measures along with examining how IWD’s experienced unmet needs, behaviors, and behavioral distress. Results found that individuals with mild to moderate dementia (n=12) were able to provide reliable self-report data about their own unmet needs (∝=.96), unmet need-related distress (∝=.98), and behavioral expressions (∝=.91). Nearly all participants identified at least one unmet need (74.8%). The highest reported unmet needs concerned health information (75.0%) and findings/arranging services (41.7%). The least reported unmet needs concerned daily living activities (16.7%) and legal/financial services (33.2%). The highest unmet needs related-distress concerned emotional support (41.7%) and the least distressing concerned daily living activities (16.7%). The most frequently reported behaviors included agitation (66.7%) and complaining/criticizing things (58.3%) while the least reported behaviors were refusing to be left alone (8.3%) and yelling/swearing (8.3%). The highest behavioral distress reported was agitation (58.3%) while the least was wandering (8.3%). Discussion will highlight how these results fit within current literature, recommendations for future research in exploring these constructs using self-report methodology, and how well-being outcomes may be impacted.

